# Use of Portable Imaging Modalities in Patients With Neurologic Disorders: A Case-Based Discussion

**DOI:** 10.7759/cureus.15841

**Published:** 2021-06-22

**Authors:** Adeel S Zubair, Anna Crawford, Anjali M Prabhat, Kevin N Sheth

**Affiliations:** 1 Neurology, Yale School of Medicine, New Haven, USA

**Keywords:** portable mri, mri, neurology, covid-19, portable ct

## Abstract

Imaging technologies have significantly improved over the past few decades and play a critical role in the diagnosis and management of patients with neurologic conditions. With the evolution of these technologies to portable versions, significant implications exist for current neurologic care as well as potential improvements for the future. This article serves to describe portable imaging technologies and their potential impact on the field of neurology highlighted through the case of a patient who presented with symptoms consistent with a stroke.

## Introduction

The primary tools for neurologists are the clinical history and physical examination. They are used to help differentiate between subtle clinical findings and to assist the clinician with localizing the lesion. Neurologists’ toolkits also include various imaging modalities. These imaging tools allow for visualization of the nervous system, identify and characterize lesions, and create treatment plans. With the coronavirus disease 2019 (COVID-19) pandemic, access to portable imaging has become even more important, both in the inpatient and outpatient settings. We present a case where portable MRI was assisted in the workup of a patient and allowed for expedited in-hospital care. This article will discuss the utility of remote imaging assessments, with portable MRI being a focus.

## Case presentation

A 69-year-old right-handed woman with a history of epilepsy secondary to a traumatic brain injury and hypertension presented to the hospital with symptoms of right-sided upper extremity weakness. She went to bed the previous night without any symptoms and upon waking noted that she was experiencing right-sided weakness so she presented to the ED.

**Figure 1:**
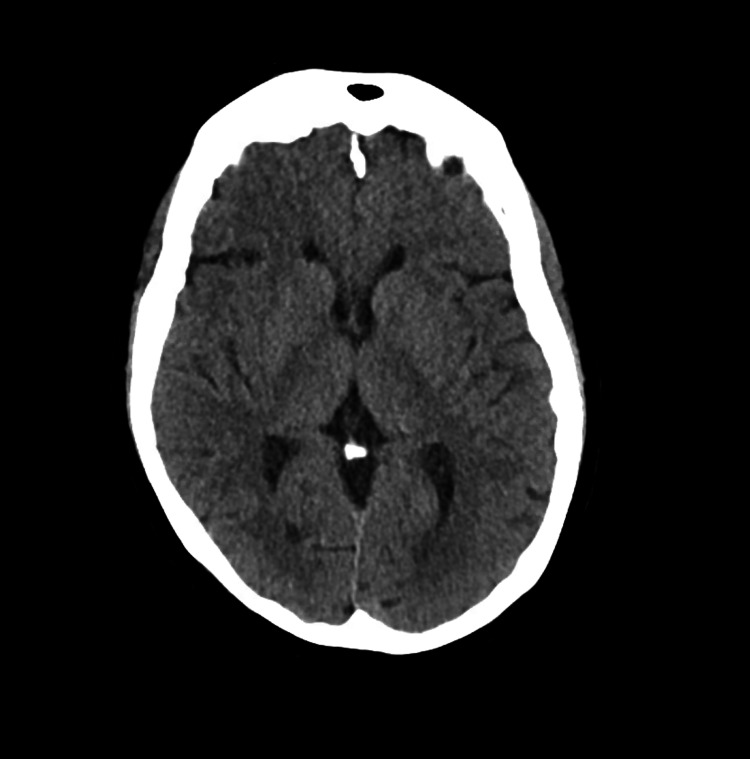
CT imaging of the head without intravenous contrast showing no evidence of any acute abnormalities.

**Figure 2:**
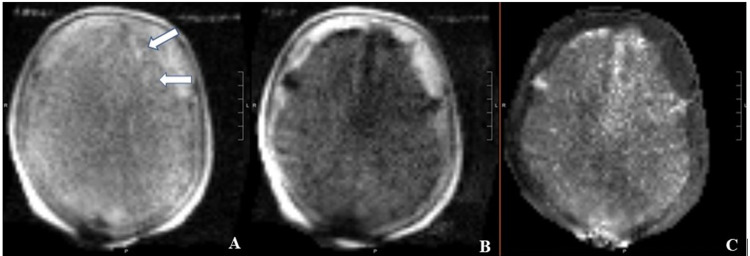
Portable MRI of the brain without intravenous contrast. Portable MRI of the brain without intravenous contrast showing evidence of scattered embolic strokes (diffusion-weighted imaging sequence [A and B] and apparent diffusion coefficient sequence [C]).

Upon presentation to the ER, the patient had a blood pressure of 140/77, a temperature of 97.7 F, a heart rate of 77, and oxygen saturation (SpO2) of 100%. A stroke code was activated and the patient was seen by the neurology team who noted right arm pronator drift and mild right-sided sensory changes with a National Institutes of Health (NIH) stroke scale score of 2. On confrontational testing, the patient had full strength except for the right upper extremity where she had weakness of the right deltoid, biceps, and triceps muscles (4/5 on the Medical Research Council scale). As part of the stroke code, a CT scan of the head without contrast was done which showed no evidence for a hemorrhagic stroke (Figure 1). The patient was outside the window for alteplase and there was no large vessel syndrome. As part of her stroke code, an MRI of the brain was ordered and the patient quickly underwent a portable MRI of the brain. The portable MRI showed evidence of scattered punctate acute infarcts within the left frontal cortex and subcortical white matter, as well as left frontal encephalomalacia/gliosis (Figure 2).

The patient was diagnosed with a potential cardioembolic stroke and risk factor modification was initiated with antithrombotic therapy (aspirin 81 mg daily). The patient completed her inpatient workup rapidly. Laboratory workup included checking hemoglobin A1c (5.4%), cholesterol (200 mg/dl, normal 140-240), and low-density lipoproteins (86 mg/dl). A transthoracic echocardiogram was done which showed an ejection fraction of 70% with evidence of a patent foramen ovale and mild-moderate mitral valve regurgitation. Portable MRI allowed for a rapid inpatient workup and helped ascertain a potential mechanism of the stroke. The patient was discharged home roughly 30 hours after initial presentation to the hospital with plans for outpatient physical and occupational therapy, close stroke follow-up, and cardiac monitoring with a 30-day Holter monitor.

## Discussion

Imaging has played a critical role in medicine since the advent of early technology, such as X-rays [1]. In neurology, the imaging modalities of magnetic resonance, CT, and ultrasound are among the most commonly used. These tools provide assistance in localizing and diagnosing lesions resulting in clinical disease manifestations. With the COVID-19 pandemic, portable imaging technology plays an important role now more than ever.

During the early days of the COVID-19 pandemic, patient transport to advanced neuroimaging was limited due to infection control concerns and, in many cases, patient instability [2]. This concern for transmission of infectious diseases applies to diseases outside of severe acute respiratory syndrome coronavirus-2 (SARS-CoV-2) as well; the arrival of multidrug-resistant bacteria and other infectious diseases often necessitates contact precautions that limit the spread of these diseases [3]. By having imaging studies in patient rooms, the exposure to other patients and hospital personnel can be limited.

Another critical role for portable imaging is in care settings where patients cannot be safely transported due to a tenuous clinical situation [4, 5]. Patients in ICUs or under close monitoring are often connected to multiple machines and tools for both monitoring and treatment. Often, such patients would benefit from conventional imaging but are unable to receive it due to the clinical dangers associated with transporting them. Imaging that can be done at the bedside can allow for an important clinical decision-making tool while also optimizing patient safety.

Portable CT scanners have been used in critical care settings for a number of years [4-7]. These studies allow for in-room assessments of patients, saving time and limiting the transfer of unstable patients. Studies have shown that these scanners are safe and provide adequate radiologic quality for diagnostic decisions [4-7]. With portable scanners becoming more common in hospitals, providers should be aware of their availability and employ tools to utilize them as frequently as possible.

More recently, portable MRI machines have also become available which allow for higher quality images when compared with CT studies [8, 9]. The current slate of portable MRI machines uses lower magnetic fields which allow them to move into the clinical environment with ease. They are safe for use without having to remove rooms of all magnetic objects and do not interfere with ICU room monitoring devices [8, 9]. In our case, the portable MRI field strength was 64 mT.

The advent of these portable imaging devices also allows for revolutionary change in the management of emergency neurologic care. Many hospitals have started to use mobile stroke unit which can contain a CT scanner in the ambulance [10-12]. This allows for earlier administration of thrombolytics by allowing for providers to rule out the presence of hemorrhage en route to the hospital. Data from these mobile stroke units can further guide patients to either a primary stroke center or a comprehensive stroke center based on the need for further diagnostic work-up and treatment [10-12].

Our case illustrates another advantage of portable imaging, namely MRI, which is faster workup and decreased hospital stays. By obtaining critical imaging early, providers can have a better understanding of the etiology of patient symptoms and craft workup more directly with the proposed cause. Faster hospital stays optimize the well-being of the patients while limiting hospital-acquired infections.

Lastly, the COVID-19 pandemic has resulted in a massive expansion of teleneurology. Currently, limitations of teleneurology include difficulty in examining patients and the ongoing necessity for presentation to hospitals or clinics for further diagnostic workup, including imaging. With the continued evolution of these portable imaging technologies, it may be possible for mobile imaging units to conduct the requested imaging studies at the patient’s residence, allowing for the strengthening of teleneurology networks. This would be critically important for patients who have limited mobility or are bed-bound and would allow for improved neurologic care in a subset of patients who can be hard to assess. 

Current limitations of portable imaging include a resolution that is not equivalent compared to their non-mobile forms. However, with improved technology and advanced computer algorithms, this can continue to improve.

Both neurology and general providers should be well versed in these portable technologies. The ability to rapidly assess patients in a multitude of different settings can lead to more efficient and practical neurologic care. Trainees who are currently starting their neurology careers will likely see these technologies play a central role in future years.

## Conclusions

Portable imaging technologies have greatly evolved over the past decade and look to be important pillars of future neurologic care. These technologies can allow for improved emergency and in-hospital neurology care. Our case describes the potential use of portable imaging technology and the benefits it can provide to patients. Future advancements of this technology may even allow for providers to order imaging tests that can be done at the patient’s house. Neurology trainees and providers should be knowledgeable about available technologies and utilize them to continue to provide high-quality care for all patients.
